# The neural and psychophysiological effects of cannabidiol in youth with alcohol use disorder: A randomized controlled clinical trial

**DOI:** 10.1038/s41386-025-02141-z

**Published:** 2025-06-11

**Authors:** Anna E. Kirkland, Brittney D. Browning, Lindsay R. Meredith, Elizabeth Robertson, Cori Herring, Rachel L. Tomko, Kevin M. Gray, Lindsay M. Squeglia

**Affiliations:** Medical University of South Carolina, Department of Psychiatry and Behavioral Sciences, Charleston, SC USA

**Keywords:** Drug screening, Addiction, Human behaviour, Drug development

## Abstract

Novel treatment evaluation for youth with alcohol use disorder (AUD) is needed. Cannabidiol (CBD), a constituent of the *Cannabis sativa* plant, may be a promising candidate pharmacotherapy due to its potential therapeutic properties and preclinical research suggesting it decreases alcohol use. Due to limited data in humans, rigorous screening of the acute neural, psychophysiological, and alcohol-related effects of CBD is indicated to assess its viability as a potential treatment for youth AUD. Using a within-subjects, randomized, double-blind, placebo-controlled design, we tested acute multi-modal effects of CBD (600 mg) in non-treatment seeking youth with AUD (*N* = 36; ages 17–22; 69% female). Outcomes included (1) glutamate+glutamine (Glx) and GABA levels in the anterior cingulate cortex measured with proton magnetic resonance spectroscopy; (2) whole-brain and a priori region-of-interest neural alcohol cue-reactivity measured with functional MRI; (3) psychophysiological response to alcohol olfactory cues measured by self-reported acute alcohol craving, heart rate variability, and skin conductance; and (4) alcohol use. No CBD-associated adverse events were observed. There were no effects of acute CBD administration, compared to placebo, on any outcomes of interest. This is the first adequately powered medication screening study for the use of CBD in youth with AUD. We did not detect significant effects of CBD on neurometabolic, neurobehavioral, psychophysiological, or alcohol use outcomes in this sample. Future studies may benefit from chronic administration to better understand substance-related effects.

Clinicaltrials.gov NCT05317546 https://clinicaltrials.gov/study/NCT05317546

## Introduction

Nearly 15% of adolescents meet diagnostic criteria for AUD by age 18 [1]. Current treatments for youth with AUD are primarily psychosocial, such as cognitive behavioral therapy, family-based therapy, and motivational interviewing [2]. Given the modest efficacy of current psychosocial treatments [3–7], pharmacotherapy has been explored as a complement to the standard of care [8]. There are limited data regarding the efficacy of pharmacotherapy in treating youth with AUD [9]. This calls for the evaluation of novel treatments specifically for youth to effectively intervene early in the AUD trajectory.

Cannabidiol (CBD) may be a promising candidate pharmacotherapy for youth with AUD. CBD is a constituent of the *Cannabis sativa* plant that has garnered attention as an alternative therapy [10]. A growing preclinical literature [11] indicates that CBD reduces alcohol consumption and motivation to drink [12–15], alcohol relapse and withdrawal [13, 16], and alcohol-related neurotoxicity [17, 18]. CBD is particularly intriguing as a treatment option for youth with AUD since it is non-intoxicating, is generally well-tolerated, and demonstrates no signal of abuse liability [19–21]. Further, young people tend to have a more positive attitude towards “natural” or “alternative” medicine [22, 23], making CBD a more appealing option than currently approved pharmacotherapies for adults with AUD.

CBD may affect alcohol consumption [24, 25] and AUD-like behaviors via its multiple brain targets that overlap with systems underlying AUD. CBD can act as an inverse agonist or negative allosteric modulator of cannabinoid receptors (CB1, CB2) [26–29] and can modulate endocannabidoid system signaling through inhibiting the enzymatic breakdown of anandamide, an endogenous cannabinoid ligand [30, 31]. CB1 and CB2 receptors are expressed throughout the mesocorticolimbic pathway, which is highly implicated in addictive behaviors (e.g., reward, decision-making, substance intake, motivation, withdrawal, and relapse) [32–35]. Thus, CBD may exert its effects on AUD symptoms through direct or indirect modulation of the endocannabinoid system. Beyond the endocannabinoid system, CBD has been shown to modulate glutamate [36, 37], gamma aminobutyric acid (GABA) [38], dopamine [39], serotonin [40, 41], and opioid [42] neurotransmission, all of which are implicated in AUD and have each been proposed as treatment targets for adult AUD pharmacotherapies [43]. The proposed neural mechanisms of action for CBD provides biological plausibilty for its potential use in AUD.

Due to the lack of data in humans, and youth in particular, it is important to rigorously screen the acute neural, psychophysiological, and substance use effects of CBD in youth with AUD prior to large scale clinical trials [44, 45]. While there are limitations to assessing in vivo neural mechanims in humans, magnetic resonance imaging (MRI) provides non-invasive techniques, such as measuring neurochemistry with proton magnetic resonance spectroscopy (1H-MRS) and alcohol cue-reactivity for a proxy of craving with functional MRI (fMRI) [46]. Along with other neurometabolites, 1H-MRS can measure glutamate and GABA levels in the brain, which are both affected by alcohol use [47], common targets for AUD medication development [48–50], and have been modulated by CBD in preclinical work [36–38]. Limited work in humans indicates that an acute dose of 600 mg of CBD can alter neurometabolite levels; individuals with psychosis [51] and austim spectrum disorder (ASD) [52] demonstrated increased glutmate levels with this 600 mg single-dose administration. Additionally, CBD adminstration resulted in decreased levels of GABA in the prefrontal cortex of individuals with ASD [52].

fMRI localizes and quantifies brain activity, allowing for a mechanistic understanding of the neural substrates affected by CBD. Specifically, fMRI alcohol cue-reactivity tasks are of interest in early medication screening studies as they can activate incentive salience and reward circuitry that underpin AUD symptomology [53]. Alcohol cue-reactivity tasks can correlate with alcohol craving [54], which is an important component to consider during AUD treatment development. Importantly, neural reactivity to alcohol cues has been modulated by previous pharmacological interventions [54, 55], making it a useful screening method during early translational studies.

Beyond the brain, CBD may exert effects on salient psychophysiological responses to alcohol cues. Psychophysiological effects of CBD can be assessed using an olfactory alcohol cue-reactivity task measuring heart rate, skin conductance, and subjective alcohol craving in response to a preferred beverage containing alcohol [56]. This in vivo task is appropriate for youth, given oral administration of alcohol to underage youth is unethical. Together, measuring the neural and the psychophysiological reactivity to alcohol using multiple sensory modalities (i.e., visual and olfactory) will help to provide a more comprehensive understanding of CBD’s mechanisms.

This study represents the first medication screening procedure designed to investigate the effects of CBD in youth with AUD. Using a randomized, placebo-controlled, crossover design, we tested the acute neurometabolic, neurobehavioral, and psychophysiological effects of CBD in non-treatment seeking youth with AUD. We hypothesized that after CBD administration, youth with AUD would have: (1) increased glutamate (as glutamate + glutamine, or Glx) and GABA levels in the anterior cingulate cortex (ACC), indicating changes in two neurotransmitter systems involved in addictive behaviors [57–59] that have been proposed as therapeutic targets of CBD [36–38]; (2) decreased alcohol cue-reactivity in reward-related neural regions [60], indicating changes in craving which is a proposed therapeutic mechanism of CBD in substance use disorders [16, 61]; and (3) lower in vivo psychophysiological response to olfactory alcohol cues [62], which may be due to the anxiolytic effects of CBD [63]. As an exploratory analysis, we also assessed if CBD impacted alcohol use behaviors up to 7-days after administration, as compared to placebo. This multi-modal approach (neuroimaging, psychophysiological response, and alcohol use assessment) was intended to provide complementary insights into the potential effects and mechanisms of CBD in youth with AUD.

## Methods

### Participants

**Fig. 1:**
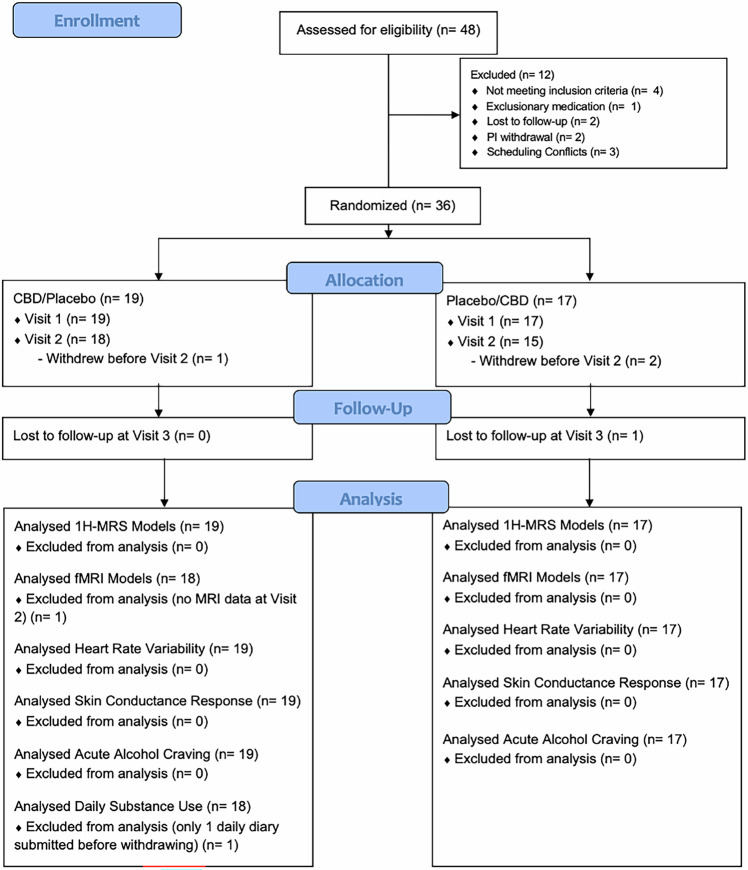
CONSORT Diagram. *CBD* cannabidiol, 1*H-MRS* proton magnetic resonance spectroscopy, *fMRI* functional magnetic resonance imaging.

48 non-treatment seeking participants (17–22 years old) were recruited (Fig. 1) from September 2022 to April 2024, and data collection was completed in June 2024. For inclusion, all participants met criteria for AUD in the past year, had at least 1 AUD symptom in the past 30 days (besides craving), and drank alcohol within 2 weeks prior to screening. See supplemental materials for exclusion criteria. Participants were recruited using a mix of approaches, including social media campaigns and in-person events.

### Consent and screening

All participants provided written informed consent/assent, and dual written permission was obtained from parents/guardians of individuals under the age of 18 years. All participants completed a centralized intake process for youth substance use studies at the Medical University of South Carolina to determine eligibility before consenting for this specific study [64]. The centralized intake process has been described in detail elsewhere [64]. In brief, the eligibility criteria for multiple studies enrolling a similar population are concurrently assessed using an IRB-approved protocol, and participants consent to share their screening data with the study in which they ultimately enroll. The Mini International Neuropsychiatric Interview (MINI) was administered to ascertain current or lifetime history of the major psychiatric disorders in DSM-5 and ICD-10 [65, 66], including past year AUD and any continuing symptoms in the 30-days per inclusion criteria. A medical clinician assessed health (e.g., current medication use) and safety to participate in the study during screening.

### Procedures

**Fig. 2:**
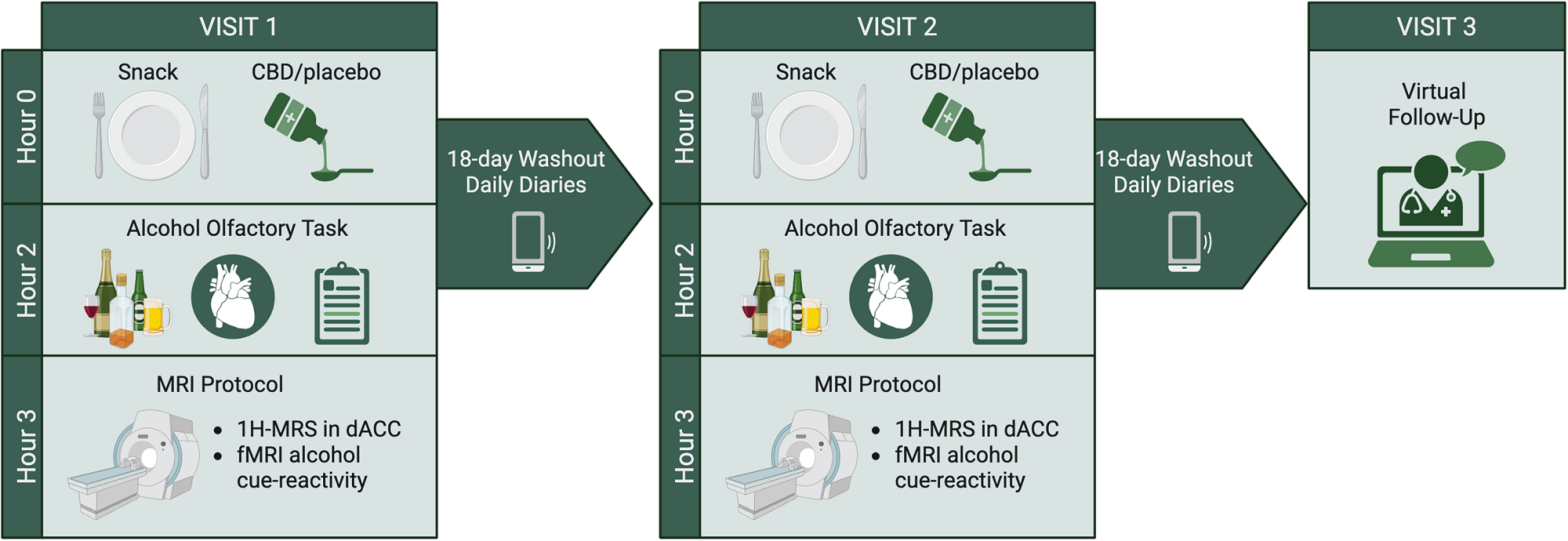
Experimental design of the clinical trial. Figure created with BioRender.com.

All procedures were approved by the local IRB and registered on Clinicaltrials.gov (NCT05317546; Fig. 2). In counterbalanced order, participants received either 600 mg of CBD (Epidiolex) oral solution [51, 52, 67, 68] or a matched placebo (sesame oil with strawberry flavoring). All participants ate a high-fat snack (70% fat content) in the presence of study staff prior to medication administration to increase the bioavailability of CBD [60]. Human laboratory and imaging procedures were conducted between 2 and 3 h after medication administration to allow for peak plasma concentration [52, 69]. Of note, the olfactory cue-reactivity task [56], 1H-MRS [70, 71], and fMRI alcohol cue reactivity [55, 72] procedures have all been previously conducted in youth. There was a minimum 18-day washout between medication administration visits [73] (terminal half-life 18–32 h after acute oral dose [74]) and before the virtual follow-up visit. See supplemental materials for more details. Over the 18-day washout periods, participants were sent daily surveys to document substance use. REDCap was used for data collection and storage [75]. Participants met with a medical clinician at every medication visit who assessed medication use, general health, and adverse events.

### Proton magnetic resonance spectroscopy

See minimum reporting standards for 1H-MRS data in Table S1 [76]. All scans were performed with a Siemens 3.0 T Prismafit MR scanner with actively shielded magnet and high-performance gradients (80 mT/m, 200 T/m-sec) with a 32-channel head coil. The dorsal ACC (dACC) voxel for 1H-MRS was placed on midsagittal T1-weighted images (Fig. S1), anterior to the genu of the corpus callosum, with the ventral edge of the voxel aligned with the dorsal edge of the genu [63], with a voxel size of (30 × 25 x 25) mm3 [63, 64]. Following FAST(EST)MAP shimming [65], single-voxel water-suppressed (water suppression bandwidth 50 Hz for Glx or 100 Hz for GABA + , spectral bandwidth 2000 Hz, 1024 spectral points) 1H-MRS spectra is acquired with the following sequences: (1) glutamate and other metabolites: SIEMENS Point Resolved Spectroscopy (PRESS) sequence: Repetition Time (TR) = 2000 ms; Echo Time (TE) = 40 ms; number of averages = 256; and (2) GABA + : SIEMENS WIP MEGA-PRESS sequence: Edit ON(OFF) = 1.90 (7.46) ppm; TR = 2000 ms, TE = 68 ms; number of averages= 160. Example spectrums are shown in Figs. S2, S3. Unsuppressed water spectra were co-acquired and scaled for partial volume effects and relaxation and used as a concentration reference. Six saturation bands (41 mm thickness) were placed 0.8 mm from each voxel face for outer volume suppression. MRS data were processed using Osprey [67], described in more detail in the supplemental materials.

### fMRI cue-reactivity task

For the alcohol cue-reactivity task [77], participants were shown pseudo-randomly interspersed images of alcohol (i.e., beer, wine, and hard liquor) and non-alcohol (e.g., soft drink, juice) beverages, visual control images (i.e., blurred images), and a fixation cross. See supplemental materials for more task details.

Functional and anatomical preprocessing was performed using FSL FEAT (v6.00, FMRIB’s Software Library), described in more detail in the supplemental materials. First-level statistical analysis was performed using the general linear model (GLM) to model task-related activity. Experimental conditions (alcohol beverage, non-alcohol beverage, blurred images, rest, and rating blocks) were modeled as explanatory variables (EVs), each convolved with a double-gamma hemodynamic response function. The main contrasts of interest were the alcohol beverage block and alcohol – non-alcohol beverage contrast. Lower-level FEAT directories were used for higher level mixed-effects analysis (FLAME 1) to estimate group-level effects (CBD vs. placebo). For whole brain analyses, cluster-based correction for multiple comparisons was performed using Gaussian random field theory with a cluster-forming threshold of Z > 3.1 and a corrected significance threshold of *p* < 0.05. For region of interest (ROI) analyses, FEATQuery was used with Z > 3.1 and a corrected significance threshold of *p* < 0.05. ROIs of interest included: the midline dACC and bilateral amygdala, caudate, insula, nucleus accumbens, and putamen [70, 78].

### Psychophysiological olfactory cue-reactivity task [56]

All participants underwent an in vivo, olfactory alcohol cue exposure procedure before the neuroimaging session. As many participants were under the legal drinking age in the United States, olfactory cues were used rather than taste cues. Prior to the task, all participants were asked to smell a candle and identify the scent (cinnamon) to ensure their ability to complete the olfactory task. Participants smelled water followed by the participant’s preferred beverage containing alcohol and apple juice (as this is not typically used as a mixer with alcohol) in a counterbalanced order for three minutes each, with a three-minute rest period in between each liquid. The contents were poured into a cup in the participant’s presence. After each beverage exposure, self-reported alcohol craving was collected via the PhenX Toolkit Alcohol Urges Questionnaire (AUQ). The AUQ consists of eight statements about the participant’s feelings and thoughts about drinking as they are completing the questionnaire (i.e., right now). The participant was asked to respond to each statement about alcohol craving via a 7-item Likert scale ranging from “strongly disagree” to “strongly agree.” Electrocardiogram (ECG), skin conductance data, and breathing rate via respiration belt were continuously recorded throughout the task using the AcqKnowledge data acquisition system (Biopac Systems, Inc., Goleta, CA). See supplemental materials for more details. The ECG data were used to create the heart rate variability (HRV) outcomes related to the sympathetic response, vagal response, their ratio (sympathetic: vagal), respiratory sinus arrhythmia (RSA), root mean square of successive differences (RMSSD), standard deviation of successive differences (SDSD), and percentage of NN50 intervals (pNN50). See Table S2 for definitions.

### Substance use

Substance use histories were assessed using a modified Timeline Follow-Back [79] (TLFB) to obtain information on typical use of alcohol, nicotine, cannabis, and other substances at screening. TLFB was completed for the 60 days prior to the screening visit to establish eligibility for the study and used as baseline covariates (details below). The following substance use variables were calculated: (1) total number of standard drinks, (2) average number of standard drinks on drinking days, (3) total number of alcohol use days, (4) binge drinking episodes (4+ drinks for females, 5+ drinks for males per day), (5) nicotine use days, and (6) cannabis use days (summed over various methods of use for nicotine and cannabis).

Over the two medication washout periods, participants reported daily substance use (i.e., alcohol, cannabis, nicotine, and other substances) through secure REDCap surveys sent via text message each morning [75, 80, 81]. See supplemental materials for more details.

To assess the acute effects of CBD, we analyzed self-reported alcohol use in the 7-days after CBD/placebo administration, inclusive of medication dose day. We selected this timeframe considering several factors. First, medication-related effects following a 1-time dose of CBD are anticipated to be short-term (e.g., half-life estimated at 18–32 h after acute oral dose). Second, youth typically engage in episodic binge drinking often occurring on or before weekends. Thus, we selected a short timeframe that would also allow for weekends to be captured, regardless of which weekday medication was administered to prevent any confounding factors. One participant was excluded, as only one daily report was completed before dropout.

### Adverse events

A thorough evaluation was conducted by a licensed medical clinician at each visit, and adverse events were coded using MedDRA terminology by body system, severity, and relatedness to study treatment.

### Statistical analysis

An a priori power analysis was conducted to ensure power to detect differences in neurometabolite levels in the dACC and fMRI alcohol cue-reactivity. See supplemental materials for more details. Given limited literature in this area, we were not able to run a power analysis for the psychophysiological olfactory task nor the substance use outcomes; this study will provide data to power future trials.

All analyses were conducted in R Statistical Software (v4.4.2; R Core Team 2024), except for the fMRI cue-reactivity models detailed above. Significance was set at alpha <0.05. The distribution of each variable was evaluated with Shapiro Wilks tests, and non-parametric tests were used for those that were not normally distributed. For 1H-MRS data quality, two sample t-tests or Wilcoxon tests were completed to assess for medication differences.

We used linear mixed effects models (lme4 package [82]), containing the main effect of medication (CBD vs. placebo), visit (visit 1 vs. visit 2), and sequence (CBD/placebo vs. placebo/CBD) to ensure the crossover design and washout period were successful. Random intercepts were included to account for individual differences. Model assumptions were checked with Performance package [83] and DHARMa package [84]. When needed, robust standard errors were used for models with heteroscedasticity [85–88]. The following covariates were considered: age, sex, 1-year AUD symptom count, 1-year AUD severity (2-3 symptoms = mild, 4-5 symptoms = moderate, and 6 or more = severe), drinks per drinking day (DPDD), total drinking days, and total binge drinking days. Covariates were tested using the likelihood-ratio test (lrtest package [89]) and included if they significantly improved model fit. For 1H-MRS models, brain tissue composition [Gray Matter: Brain Matter or GM:BM defined as GM/(GM + WM)] was included as a covariate.

For the olfactory task, models for AUQ, HRV, and SCR were first run as described above with additional cue (water, apple juice, or alcohol) and cue-by-medication interaction terms. The interaction term was included for AUQ models, but not for HRV or SCR, as cues did not elicit significantly different responses in HRV and SCR.

Exploratory daily diary analyses focused on alcohol use collected between medication doses. For the outcome, daily number of drinks, a mixed-effects, zero-inflated negative binomial regression model was used instead (glmmTMB package) due to a substantial number of zeros (i.e., non-drinking days).

Our registered main outcomes were 1H-MRS levels of Glx and GABA + ; fMRI alcohol cue-reactivity; and the physiological outcomes from the olfactory cue-reactivity lab-based paradigm. Thus, the daily diary analyses and additional neurometabolites (tNAA, tCho, tCr, and mI) were exploratory (NCT05317546).

## Results

### Participants

36 participants were randomized (age 17.6–22.8). Almost 70% of the participants reported being biologically female at birth, 66.7% of the full sample identified as being a woman, and 86% of participants reported being white. For past 1-year AUD severity criteria, 55.6% met for mild AUD, 27.8% met for moderate AUD, and 16.7% met for severe AUD. A lower number of participants met for cannabis use disorder (44.4%). Over half the sample reported current medication use at screening (66.1%), and there were 13 reports of changes to medication use at Visit 1 (*n* = 5), Visit 2 (*n* = 6), or Visit 3 (*n* = 2). Medication additions were mainly antibiotics, cold medicine, or allergy medicine (53.4%), ADHD (15.4%), skin conditions (7.7%), weight loss medication (7.7%), or changes to dosing of medications already reported (15.8%). See Table 1 for complete participant demographics.

**Table 1:** Demographics of randomized participants (*N* = 36).

Randomized Participants (*N* = 36)			
Demographics	Mean	SD	Range
Age	20.47	1.46	17.59–22.83
Most Recent Grade Point Average	3.62	0.33	2.98–4.00
	***N***	**%**	
Biological Sex			
Female	25	69.4%	
Male	11	30.6%	
Gender Identity			
Woman	24	66.7%	
Man	12	33.3%	
Ethnicity			
Not Hispanic or Latino/a	34	94.4%	
Hispanic or Latino/a	2	5.6%	
Education Completed			
10^th^ Grade	1	2.8%	
High School	5	13.9%	
Some College	23	63.9%	
Bachelor’s Degree	7	19.4%	
Race			
White	31	86.1%	
Black or African American	3	8.3%	
Asian American	1	2.8%	
Multiracial	1	2.8%	
**DSM-5 Psychiatric Diagnoses**	***N***	**%**	
Current Alcohol Use Disorder	36	100.0%	
Mild	20	55.6%	
Moderate	10	27.8%	
Severe	6	16.7%	
Current Cannabis Use Disorder	16	44.4%	
Mild	7	19.4%	
Moderate	8	22.2%	
Severe^a^	1	2.8%	
Current Major Depressive Episode	3	8.3%	
Lifetime Major Depressive Episode	14	38.9%	
Current Panic Disorder	4	11.1%	
Lifetime Panic Disorder	7	19.4%	
Current Generalized Anxiety Disorder	6	16.7%	
Current Social Phobia	3	8.3%	
Current Obsessive-Compulsive Disorder	0	–	
Current Agoraphobia	2	5.6%	
Current Attention Deficit /Hyperactivity Disorder (ADHD)	4	11.1%	
ADHD Combined Type	3	8.3%	
ADHD Inattentive Type	0	–	
ADHD Compulsive Type	1	2.8%	
Current Posttraumatic Stress Disorder	0	–	
**Current Medication**^**b**^	**N**	**%**	
Any Medication	22	61.11%	
Birth Control	10	45.45%	
Vitamins/Supplements	10	45.45%	
Anxiety or Depression	10	45.45%	
Allergies	7	31.82%	
Skin Condition	3	13.64%	
Pain Relief	2	9.09%	
Antibiotics	2	9.09%	
ADHD	2	9.09%	
Bipolar Disorder	1	4.55%	
Asthma	1	4.55%	
Steroids	1	4.55%	
Crohn’s Disease	1	4.55%	
Vertigo	1	4.55%	
HIV Prevention	1	4.55%	
**Recent Substance Use and Mental Health**	**Mean**	**SD**	**Range**
Age when first consumed an alcohol-containing beverage	15.60	1.94	12.00–20.75
Largest alcohol use (standard drinks) per occasion in past year	10.06	4.45	5–26
Rutgers Alcohol Problems Index Score	5.61	4.62	0–17
Alcohol Use Past 60 Days			
Drinking Days	13.58	5.83	3–29
Total Drinks	62.28	44.12	10.25–246.0
Binge Drinking Days	6.75	5.18	0–17
Drinks Per Drinking Day	4.66	2.56	1.14–13.67
Any E-cigarette Use Past 60 Days (%)	16	44.4%	
Any Cannabis Use Past 60 Days (%)	25	69.4%	
Pittsburgh Sleep Quality Index Score	5.58	2.25	2–10
Generalized Anxiety Disorder 7-Item Score	7.11	4.77	0–16
Patient Health Questionnaire-9 Score	5.33	3.92	0–15

^a^One participant met criteria for past year severe CUD, but had not used cannabis in the past 60-days and had a negative UDS, and therefore was included in the sample.

^b^Current medications collection and categorized by a medical clinician.

### 1H-MRS

There were no significant medication-related differences for any neurometabolite levels in the dACC (Fig. S4, Table 2). There were significant effects for past 1-year AUD symptom count, where high symptom counts related to lower levels of Glx (*B* = −0.23; *p* < 0.01) and tCho (*B* = −0.07; *p* < 0.01). More past-60-day binge drinking days at screening corresponded with lower levels of GABA+ (B = −0.02; *p* = 0.02). Finally, tNAA was related to brain tissue composition (defined by GM:BM; B = −8.86; *p* < 0.001). Tables S3, S4 show the metabolite descriptives, tissue composition, and data quality metrics. The data quality metrics were high and the CoV was low, indicating overall good data quality. There were no significant differences between CBD compared to placebo for any data quality or tissue composition outcomes, indicating consistency between CBD and placebo scans.

**Table 2:** Dorsal Anterior Cingulate Cortex Neurometabolite Levels, Linear Mixed Effects Model (*n* = 36).

Metabolite	Term	Beta Estimate	Std. Error	T-Stat	df	*P*-value
Glx	Intercept	20.68	2.81	7.36	41.28	0.00
	Medication	−0.18	0.18	−0.99	31.05	0.33
	Sequence	−0.33	0.31	−1.07	31.40	0.29
	Visit	−0.09	0.18	−0.51	31.57	0.62
	GM:BM	−0.62	3.99	−0.16	40.95	0.88
	**1-Year AUD Symptom Count**	**-0.23**	**0.08**	−**3.07**	**30.43**	**<0.01**
GABA+	Intercept	5.13	0.76	6.77	39.84	0.00
	Medication	−0.02	0.07	−0.33	34.20	0.75
	Sequence	0.02	0.08	0.24	32.96	0.81
	Visit	−0.03	0.07	−0.46	34.59	0.65
	GM:BM	−1.06	1.08	−0.99	38.62	0.33
	**Binge Drinking Days**	**−0.02**	**0.01**	**−2.45**	**35.17**	**0.02**
tNAA	Intercept	25.51	1.64	15.53	40.90	0.00
	Medication	0.04	0.15	0.25	34.31	0.80
	Sequence	−0.08	0.16	−0.47	33.55	0.64
	Visit	0.07	0.16	0.43	34.75	0.67
	**GM:BM**	**−8.68**	**2.31**	**−3.76**	**39.06**	**<0.001**
tCho	Intercept	4.24	0.75	5.69	51.95	0.00
	Medication	0.01	0.03	0.33	28.13	0.75
	Sequence	−0.13	0.09	−1.40	30.25	0.17
	Visit	−0.02	0.03	−0.55	29.02	0.59
	GM:BM	−0.79	1.06	−0.75	53.07	0.46
	**1-Year AUD Symptom Count**	**−0.07**	**0.02**	**−3.27**	**29.59**	**<0.01**
tCr	Intercept	13.35	1.68	7.93	46.65	0.00
	Medication	−0.06	0.09	−0.62	30.95	0.54
	Sequence	−0.11	0.18	−0.59	32.96	0.56
	Visit	−0.04	0.09	−0.44	31.60	0.66
	GM:BM	−1.62	2.39	−0.68	46.15	0.50
mI	Intercept	12.04	2.15	5.60	45.96	0.00
	Medication	−0.09	0.13	−0.70	32.33	0.49
	Sequence	0.10	0.23	0.43	34.02	0.67
	Visit	−0.05	0.13	−0.36	32.92	0.72
	GM:BM	−1.33	3.05	−0.44	45.30	0.66

*Glx* glutamate + glutamine, *tNAA* total N-acetylaspartate, *tCho* total choline-containing metabolites, *tCr* total creatine-containin metabolites, *mI* myo-Inositol, *GABA* *+*  GABA plus macromolecules, *GM:BM* defined as GM/(GM + WM), Medication defined as CBD (reference) or placebo, Sequence defined as CBD/Placebo (reference) or Placebo/CBD, Visit defined as neuroimaging Visit 1 (reference) or Visit 2, Binge Drinking Days from 60-day TLFB at screening, 1-Year AUD Symptom Count defined as number of endorsed AUD symptoms in the past year on the MINI at screening.

### fMRI alcohol cue-reactivity task

Neither whole brain analyses nor ROI analyses indicated any difference in neural reactivity to alcohol cues (alcohol cues alone, alcohol – non-alcohol beverage cues) during acute CBD administration compared to placebo (Z > 3.1, *p* > 0.05).

### Psychophysiological olfactory cue-reactivity task

**Fig. 3:**
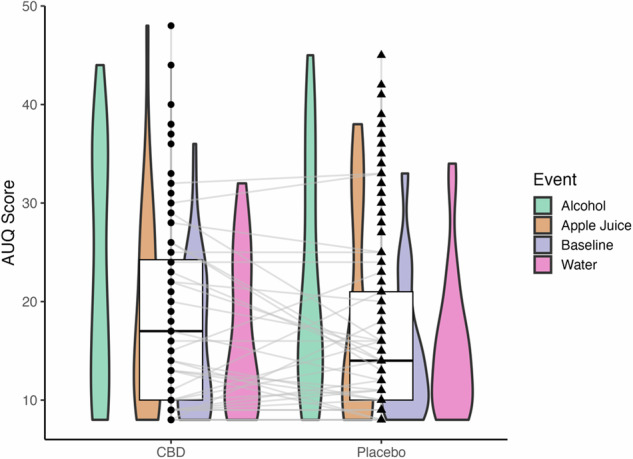
AUQ scores across cues during the olfactory cue-reactivity task. While there were no significant differences between CBD and placebo on the AUQ, the olfactory cue type did impact the outcome, where alcohol cues were related to higher reported craving (p’s<0.001). Black dot/triangle = individual AUQ response collapsed across cue condition.

For the AUQ models assessing acute alcohol craving during the olfactory cue-reactivity task (descriptives in Table S5), the cue term was significant and retained in the model (Table S6). There were no significant effects of CBD on AUQ scores, nor was there a significant cue-by-medication interaction (Fig. 3). This indicates the olfactory alcohol cues were associated with higher reported acute craving, which was not affected by CBD as compared to placebo.

For the HRV and SCR models assessing physiological response during the olfactory cue-reactivity task, the cue term was insignificant and not retained in the model. Instead, HRV and SCR across the full task was assessed. There were no significant medication effects for any HRV (Table S7-8, Fig. S5) or SCR outcomes (Table S9-10, Fig. S6**)**.

### Alcohol Use 7-days after CBD/placebo administration

On average, participants consumed alcohol on 30% of days in the week following each medication dose (descriptives in Table S11). Medication condition did not significantly predict daily number of drinks consumed (*p* = .261). The covariate, baseline DPDD was associated with greater daily drinking (*p* < .001) (Table S12).

### Safety

No adverse events or serious adverse events were reported in relation to CBD during the study, supporting CBD’s safety profile in youth with AUD.

## Discussion

CBD is often marketed and purported to treat psychiatric disorders, including alcohol and other substance use disorders, despite a lack of randomized controlled trials supporting this claim [63]. The present study is the first medication screening procedure investigating the neurometabolic, neurobehavioral, psychophysiological, and alcohol use effects of an acute dose of CBD (600 mg) compared to placebo in youth with AUD. Consistent with previous studies, the safety profile of CBD was supported. We hypothesized that CBD would impact glutamatergic and GABAergic systems, two neurotransmitters involved in addictive behaviors [57–59] that have been proposed as therapeutic targets of CBD [36–38]; decrease alcohol-cue reactivity in reward-related neural regions through reductions in craving [16, 61]; and lower in vivo psychophysiological response to olfactory alcohol cues, possibly due to the anxiolytic effects of CBD [63]. However, there were no significant effects of CBD, compared to placebo, across the multi-modal methods (neural, psychophysiological, and alcohol use). The null effects should be interpreted within the constraints of the study design and can help inform future research assessing more clinically relevant effects of CBD in youth with AUD, such as chronic dosing, inter-individual variability of CBD’s bioavailability, and the use of more salient cue-reactivity tasks.

Overall, our results contradict recent CBD studies in samples of adults with AUD. Zimmermann et al. [60] found that a single 800 mg dose of CBD in adults with AUD (*N* = 28, average age = 35.8) resulted in lower alcohol-cue activation in the nucleus accumbens and lower self-reported craving compared to placebo [60]. Similarly to the present study, their procedures were conducted 3 h after CBD or placebo administration. However, their parallel group design utilized a combined stress- and alcohol- cue exposure task that took place outside of the scanner and before the fMRI alcohol cue-reactivity task, which may have primed craving and neural reactivity. They also used a different formulation of CBD ( > 99.8% synthetic CBD) at a slightly higher dose and assessed blood levels of CBD, which was importantly related to outcomes. Hurzeler et al. found significant effects of 3-days of 800 mg of CBD compared to placebo (*N* = 22, average age = 29.0) on psychophysiological, anxiety, and alcohol craving to alcohol cues (compared to juice) outside the scanner and neural response to visual alcohol cues inside the scanner [62, 90]. Both studies implemented the outside scanner cue reactivity tasks in a bar setting, which might increase response due to environmental cues. The differences from our study may be a result of methods (e.g., dosing, stimuli, setting), age, AUD severity/duration, or other demographics.

The lack of neural effects of CBD may be indicative of sample characteristics, such as age and AUD severity. Our previously published work suggests that youth who use alcohol may not incur, or at least not at the same degree, the neurometabolic effects that are seen in adults with alcohol misuse or AUD [47, 70]. Specifically, we previously did not find differences in neurometabolites within the ACC of youth who use alcohol, or in the sub-sample of youth with AUD, compared to youth who did not use alcohol [70]. This contradicts findings in adults that suggests lower levels of both GABA and NAA in the ACC related to alcohol use [47]. Our previously reported sample [68] was younger (17–19 years old; average age 18.8) than the currently reported sample (17–22 years old; average age 20.5) and was not required to meet criteria for AUD for inclusion. The differing sample characteristics are of interest considering the current study found lower levels of Glx and tCho are related to higher past 1-year AUD symptom count and lower GABA+ levels were related to higher binge drinking days. The latter has also been shown in college students who report binge drinking [91]. This suggests that alterations in neurometabolite levels may begin to develop in early adulthood (e.g., after the age of 19). If this alcohol-related developmental effect is true, then 1H-MRS may not be granular enough to capture the effects of CBD, or other medications [71], on neurometabolite levels in youth.

The fMRI alcohol-cue reactivity task has a more established literature in youth who use alcohol; however, the findings have been mixed in terms of the neural effects of alcohol use [55, 70, 72, 92–96] and the potential to use this task in medication screening paradigms [78, 97]. We did not see an effect of CBD on neural reactivity to alcohol cues or the contrast of alcohol – non-alcohol beverage cues using both a whole-brain approach and an a priori ROI approach focused on brain regions known to underpin AUD. Our null fMRI alcohol cue-reactivity findings and self-reported alcohol craving via the AUQ contradict the above mentioned Zimmermann et al. trial [60]. It is possible that the images presented in the alcohol-cue reactivity task do not elicit strong enough craving or appetitive responses in youth with AUD, contributing the null findings. Future studies may want to investigate these effects in a larger sample that covers a broader age range, explores different doses of CBD, and employs stimuli that may be more relevant for modern day youth.

The physiological response (HRV and SCR) during the olfactory cue-reactivity task was not impacted by CBD administration. This may be due to the lack of task effects for HRV and SCR, where there were no physiological differences noted between baseline, water, apple juice, or alcohol exposure. Even though the task presented in this study has been conducted in youth populations previously [56], and the task was individualized to the participant based on their preferred alcoholic beverage, our findings suggests that the olfactory cues presented were not salient enough to illicit a physiological response in youth with AUD. Tasks with multi-system or more salient cues, such as taste [98], may be better situated to measure changes in physiological responses due to medication. Given the sample included youth under the legal drinking age for the US, we were not able to incorporate taste cues, which might be the most salient cues for this age. Taste cues have been associated with increased craving, desire to drink, reactivity to olfactory cues, and neural activation [99, 100]. Interestingly, we did see cue-induced changes in self-reported acute alcohol craving via the AUQ, where alcohol cues were related to higher craving. Previous work has also shown increases in self-reported craving after olfactory exposure to beer cues in adolescents with AUD [101]. It is unclear if this is a true response or a demand characteristic [102, 103]. Adaptations to the olfactory cue-reactivity task should be considered before utilizing it in future youth AUD studies.

The findings presented need to be interpreted within the strengths and weakness of this study. In terms of strengths, this study was a rigorously designed within-subjects crossover design that allows for stronger statistical power. We employed a strict time-lock protocol to collect data when CBD’s bioavailability should have been peaking [52, 69]. We carefully chose the acute 600 mg dose of CBD based on existing literature showing neural and clinical effects in a variety of populations, including schizophrenia, autism spectrum disorder, anxiety, and healthy control groups [20, 51, 63, 67, 68, 104–109]. We used up-to-date acquisition, processing, and analysis techniques (e.g., Osprey) that allowed for thorough visual and data-driven quality checks for all data. Consequently, our data were very high quality, and few participants had to be excluded from analyses (with exception of the olfactory cue-reactivity task SCR outcomes). We also used multi-modal methods that allowed for the investigation of several neural, biological, and behavioral factors that influence AUD symptomology. Importantly, the safety profile of CBD in this study was excellent, with no reported adverse events related to the medication. This will be important for future studies utilizing chronic dosing of CBD.

In terms of weaknesses, there is limited research in this area which made concluding power and sample size difficult. We based our power analysis on extant research that administered an acute dose 600 mg of CBD in samples that were clinically and demographically different from the present study (e.g., schizophrenia for 1H-MRS) [51] or used different fMRI procedures (e.g., resting state or task based) [20, 51, 67, 68, 104–109], which may inform the null findings. The data presented on each outcome can now be used to calculate well-powered sample sizes in youth with AUD. Unlike the Zimmermann CBD study [60], we did not quantify blood CBD levels to confirm bioavailability. This may be critical in future CBD research, as other studies have reported high levels of inter-participant variability in CBD plasma levels which were not related to physical characteristics [60]. We did carefully plan and time-lock the protocol to peak CBD time window according to existing data [52, 69] and used a standardized high-fat snack in maximize bioavailability of CBD [110]; however, confirmation of blood CBD levels would be ideal. Future work will benefit from a dose-response study in youth with AUD. We also focused on an acute dose (600 mg) of CBD rather than longitudinal dosing, which might be critical in this age range to detect substance use or other clinically meaningful effects [11, 111]. Additionally, the cue-reactivity tasks used may present ineffective cues for youth, who may be more reactive to mood enhancement, social cues, or environmental stimuli related to alcohol [103]. The fMRI alcohol cue-reactivity task relies on standard images that may not induce craving or appetitive responses in youth, limiting the ability to observe medication effects. Indeed, previous work from our group using this task has shown minimal differences in neural reactivity between youth who use alcohol heavily and a sub-group with AUD compared to controls [70], while other work has shown neural reactivity using this task in youth [72]. The olfactory cues were also not salient enough to induce physiological changes between beverage types; however, we did see increases in self-reported craving that were not impacted by CBD. Taste may be a more impactful cue [98–100], so future studies should try to power their samples to allow for taste reactivity assessment in individuals who can legally consume alcohol. Finally, our sample was very homogenous with high representation of white females which may limit the generalizability to more diverse youth or those with higher severity of AUD. This warrants further research.

## Conclusion

This medication screening study was a first attempt at understanding the acute effects of CBD, compared to placebo, in non-treatment seeking youth with AUD. While we employed a variety of methods, CBD was not related to any neural, physiological, psychological, or behavioral change. These findings should not be interpreted as conclusive regarding of CBD’s potential therapeutic role within this population. Rather, longitudinal studies that incorporate biological markers of CBD absorption and studies designed to capture potential changes in substance use behaviors may provide critical additional insights in the overall effort to rigorously develop and evaluate candidate treatments for youth with AUD.

## Supplementary information

Supplemental Materials

## Data Availability

All data are available through the NIAAA Data Archive.
