# Supplementary material for: The neural and psychophysiological effects of cannabidiol in youth with alcohol use disorder: A randomized controlled clinical trial
Source: Neuropsychopharmacology. 2025 Jun 11;50(10):1482–92. doi: 10.1038/s41386-025-02141-z (PMC12339998; doi:10.1038/s41386-025-02141-z)
Supplement: Supplementary file 1 — Supplemental Materials [file 41386_2025_2141_MOESM1_ESM.docx]

**Supplemental Materials**

Exclusion criteria included: (1) significant or acutely unstable medical, psychiatric, or substance use problems (e.g., current manic episode, positive psychotic symptoms, severe eating disorder, severe opioid use disorder); (2) significant risk of homicide or suicide; (3) currently enrolled in or acutely seeking treatment for AUD or any other SUD; (4) pregnant, trying to become pregnant, or breastfeeding; (5) known allergy or intolerance to CBD; (6) current use of CBD or any supplement containing CBD in the past 2 weeks; (7) history of a serious medical or neurological problem that could affect neural response or brain development; (8) non-correctable visual or hearing problems; (9) MRI contraindications (e.g., braces, claustrophobia, irremovable metal implants or piercings); (10) acute drunkenness or consumption of alcohol within 12 hours of visit; (11) ≥10 on the Clinical Institute Withdrawal Assessment for Alcohol (CIWA-Ar); (12) severe cannabis use disorder (CUD); and (13) concurrent medications with potential drug-drug interactions with CBD, including CYP3A4 and CYP2C19 substrates, inhibitors, and inducers, as well as CYP2C8/9 substrates.

***CBD Medication Information***

PI Squeglia received an FD exemption for using Epidiolex (CBD) with adolescents who meet criteria for alcohol use disorder (IND161500). Epidiolex (CBD) was precured by the research team and be dispensed by the Medical University of South Carolina Investigational Drug Service (IDS). The matched placebo liquid was also compounded and dispensed by the MUSC IDS. The research team picked up syringes with the blinded liquid from IDS prior to the visit. The randomization schedule was created by a team statistician and was stratified by sex at birth.

The suggested maximum target daily dosing for children with epilepsy is 25 mg/kg/day; therefore, even for a low-weight adolescent, the proposed single dose is well within target range of tolerability. A previous within-subjects study in adults with autism spectrum disorder used an acute oral dose of CBD (600 mg) and successfully prevented carry-over effects with a 13-day washout period [1]. Further, a single dose of 600 mg has been shown to modulate brain metabolite levels measured with MRS [1, 2] and blood oxygen level dependent (BOLD) signal measured with fMRI [3-6] as compared to placebo in clinical populations (autism spectrum disorder, clinical high risk for psychosis, and psychosis) and healthy controls.

The metabolism and absorption of CBD can be modulated by food intake, with a 4-to-5 fold increase in bioavailability after a high-fat meal [7]; thus, all participants will eat a standardized high-fat snack with each medication administration.

**Table S1**: MRSinMRS checklist for a single voxel ^1^H-MRS study [71]

| 1. Hardware |  |
| --- | --- |
| a. Field strength [T] | 3 T |
| b. Manufacturer | Siemens |
| c. Model (software version if available) | Prisma Fit |
| d. RF coils: nuclei (transmit/receive), number of channels, type, body part | 32 channel head coil |
| e. Additional hardware | N/A |
| 2. Acquisition |  |
| a. Pulse sequence | SIEMENS Point Resolved Spectroscopy (PRESS) sequence  SIEMENS WIP MEGA-PRESS sequence |
| b. Volume of Interest (VOI) locations | Dorsal Anterior Cingulate Cortex (dACC) |
| c. Nominal VOI size [cm^3^, mm^3^] | 30 x 25 x 25 mm^3^ |
| d. Repetition Time (TR), Echo Time (TE) [ms, s] | PRESS: Repetition Time (TR) = 2000 ms; Echo Time (TE) = 40 ms  MEGA-PRESS: Edit ON(OFF)= 1.90 (7.46) ppm; TR=2000 ms, TE= 68 ms |
| e. Total number of Excitations or acquisitions per spectrum | PRESS: 256  MEGA-PRESS: 160 |
| f. Additional sequence parameters (spectral width in Hz, number of spectral points, frequency offsets) | Spectral bandwidth 2000 Hz, 1024 spectral points |
| g. Water Suppression Method | PRESS: Water Sat; 50Hz, 90 deg  MEGA-PRESS; Water water suppr; 100Hz; 90 deg |
| h. Shimming Method, reference peak, and thresholds for “acceptance of shim” chosen | FASTEST-MAP |
| i. Triggering or motion correction method  (respiratory, peripheral, cardiac triggering, incl. device used and delays) | N/A |
| 3. Data analysis methods and outputs |  |
| a. Analysis software | Osprey 2.6.5 |
| b. Processing steps deviating from quoted reference or product | MEGA-PRESS: recommended parameters were used to optimize GABA+ processing (metabolite fit range: 0.5 to 4.0 ppm; water fit range: 2.0 to 7.4 ppm; knot spacing= 0.55; MM09 hard modelling) |
| c. Output measure  (e.g. absolute concentration, institutional units, ratio) | Absolute concentration derived using fully tissue-and-relaxation-corrected molal concentration estimates |
| d. Quantification references and assumptions, fitting model assumptions | Standard Osprey processing |
| 4. Data Quality |  |
| a. Reported variables  (SNR, Linewidth (with reference peaks)) | Signal-to-noise ratio (Cr SNR; ratio between amplitude of Cr peak and standard deviation of detrended noise) and linewidth for Cr [full-width half-maximum (FWHM) of single-Lorentzian fit to Cr peak] and water (H2O; FWHM of single-Lorentzian fit to H2O reference peak) |
| b. Data exclusion criteria | No subjects excluded based on the Cr SNR was >3 standard deviations away from the group mean or if the linewidth was >11 Hz |
| c. Quality measures of postprocessing Model fitting (e.g. CRLB, goodness of fit, SD of residual) | Measures described in 4a |
| d. Sample Spectrum | Supplementary Materials Figure S2-3 |


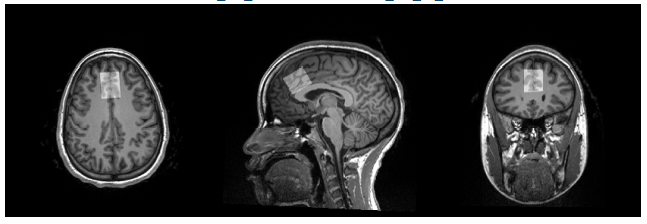


**Figure S1.** Example of the dACC voxel placement on T1 image.


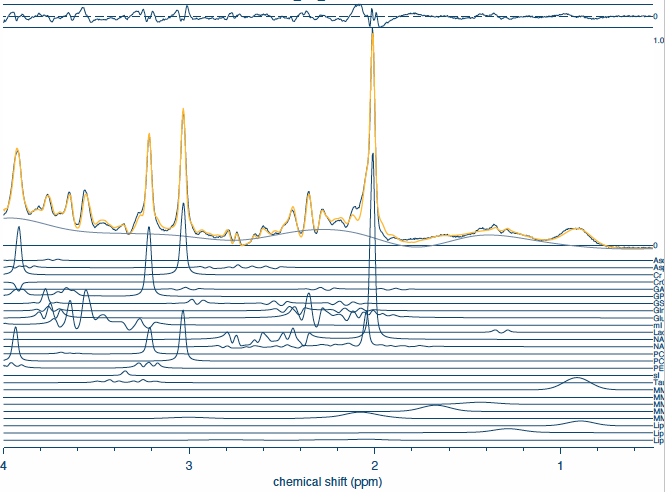


**Figure S2.** Example PRESS spectrum fit.


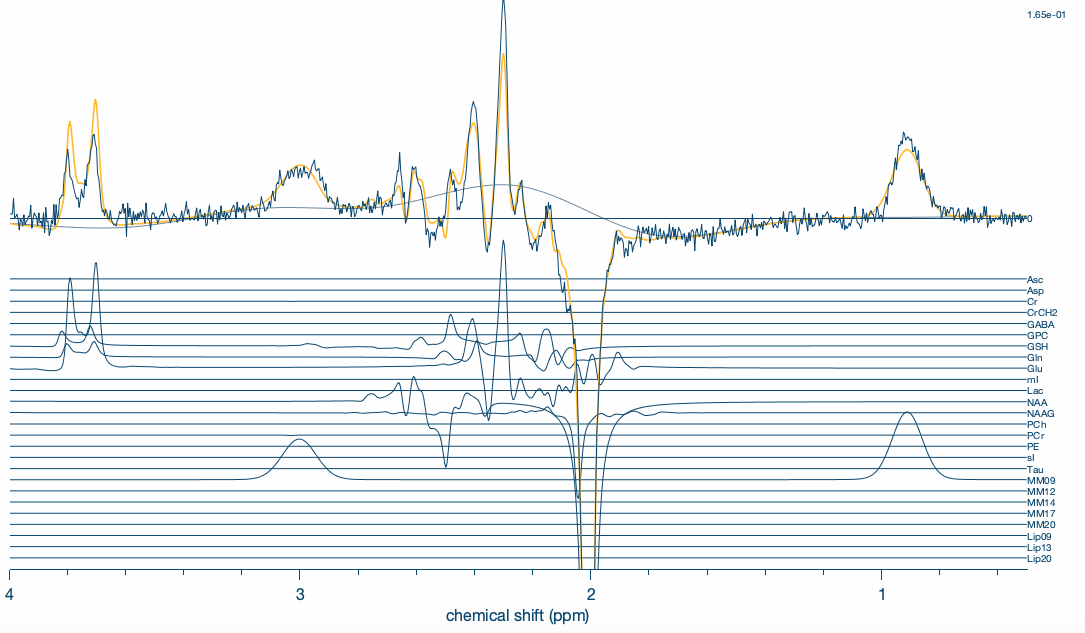


**Figure S3.** Example MEGA-PRESS spectrum fit.

***Proton Magnetic Resonance Spectroscopy****.* A high-resolution anatomical scan (magnetization prepared rapid gradient echo; MPRAGE) was acquired, to allow subsequent registration to region-of-interest (ROI) definition (parameters: repetition/echo time (TR/TE) = 2250/4.18 ms; flip angle (FA)= 9°; field of view (FOV)= 256 mm2; voxel size= 1 mm2; 176 contiguous 1-mm-thick slices). The 1H-MRS protocol was based on previously published methods [61, 62].

Osprey is an open-access, all-in-one MATLAB-based software. Standard parameters were used for Glx processing (metabolite fit range: 0.2 to 4.2 ppm; water fit range: 2.0 to 7.4 ppm; knot spacing= 0.4), while recommended parameters were used to optimize GABA+ processing (metabolite fit range: 0.5 to 4.0 ppm; water fit range: 2.0 to 7.4 ppm; knot spacing= 0.55; MM09 hard modelling) [68]. T1-weighted images were segmented using SPM12 [69] within Osprey, and voxel tissue fractions were saved [grey matter (GM), white matter (WM), and cerebrospinal fluid (CSF)]. Metabolite quantifications were derived using fully tissue-and-relaxation-corrected molal concentration estimates [70]. The primary metabolites of interest were Glx and GABA+. Secondary metabolites of interest were Glu, tNAA, tCho, tCr, and mI.

Osprey provides data quality outcomes, including signal-to-noise ratio (Cr SNR; ratio between amplitude of Cr peak and standard deviation of detrended noise) and linewidth for Cr [full-width half-maximum (FWHM) of single-Lorentzian fit to Cr peak] and water (H2O; FWHM of single-Lorentzian fit to H2O reference peak). All spectra were visually inspected for artifacts by the first author. Cases were excluded if the Cr SNR was >3 standard deviations away from the group mean or if the linewidth was >11 Hz. Additionally, the coefficient of variation (COV) was determined (standard deviation divided by the mean, where lower values indicated higher quality data) [71]. 32 participants had complete 1H-MRS data (n=3 dropped out before visit 2; n=1 without MRI data at visit 2), but all participants were retained in the models.

***fMRI Cue-Reactivity Task****.* The beverage stimuli were selected from a normative set, supplemented with images from advertisements, and matched by color, hue, and complexity. Blurred images and the fixation crossed trials are used as contrasts to evaluate attention and non-alcohol specific effects. Stimuli are presented in six 120-s epochs, each consisting of four 24-s blocks of an image type (one block each of alcohol, non-alcohol control, and fixation). Each block is followed by a 6-s washout period, allowing the hemodynamic response from the previous block to decline before the next was presented. A 12-m gradient-echo EPI sequence was acquired (parameters: repetition/echo time (TR/TE)= 2200/35 ms; flip angle (FA)= 90°; field of view (FOV)= 220 x 220 mm; voxel size= 3.44 x 3.44 mm; 32 contiguous 4-mm-thick slices).

The preprocessing pipeline included the following steps: non-brain tissue was removed from both functional and structural images using the Brain Extraction Tool (BET), MCFLIRT to correct for head motion, functional images were spatially smoothed using a Gaussian kernel with a full-width at half-maximum (FWHM) of 5 mm, a high-pass temporal filter of 100 second was applied to remove low-frequency drift, functional images were first registered to the high-resolution structural images and high-resolution structural images were registered to the MNI152 2-mm standard space template using a linear transformation with 12 degrees of freedom. At the first level, temporal derivatives were included to account for timing variability, and motion parameters were added as confound regressors. Outputs from the first-level analysis included contrast maps corresponding to task conditions of interest. ROIs were assessed using anatomically label ROIs from the Harvard-Oxford Cortical and Subcortical Structural Atlases provided in FEATQuery. 31 participants had complete, usable data (n=3 dropped out before visit 2; n=1 without MRI data at visit 2; n= 1 with head motion at visit 1).

***Psychophysiological Olfactory Cue-Reactivity Task****.* ECG was measured using three disposable electrodes: one electrode on the right clavicle, one on the left lower rib, and a ground electrode on the left clavicle. Skin conductance was measured using two Ag/AgCl electrodes filled with isotonic gel, placed on the palmar surface of the right hand. ECG and skin conductance data were originally sampled at 2000 samples per second (2000 Hz). For analysis, EKG and skin conductance data were resampled to 500 Hz to optimize computational efficiency while preserving signal fidelity. 32 participants had usable HRV data (n=3 dropped out before visit 2; n=1 had unusable data), but all participants were retained in the models. For skin conductance responses (SCR), the total number, mean, standard deviation, and median amplitudes were assessed, as well as the peak-to-peak interval (defined as time from onset of SCR to peak amplitude). 24 participants had complete, usable SCR data (n=3 dropped out before visit 2; n=9 had unusable data), but participants with at least 1 usable visit were retained in the models.

**Table S2. Heart-Rate Variability (HRV) Terms and Definitions**

| Term | Definition | Related System |
| --- | --- | --- |
| Sympathetic Response | Represented indirectly through low-frequency power (0.04–0.15 Hz) in frequency-domain analysis. | Reflects a mix of sympathetic and parasympathetic activity, though more weighted toward sympathetic. |
| Vagal Response | Represented by high-frequency (HF) power (0.15–0.4 Hz) in frequency-domain analysis. | Directly related to parasympathetic (vagal) tone; sensitive to respiratory influences. |
| Sympathetic: Vagal Ratio | The ratio of low-frequency to high-frequency power, derived from spectral HRV analysis. | Higher values suggest increased sympathetic activity or reduced vagal activity. |
| Respiratory Sinus Arrhythmia (RSA) | Calculated using peak-to-valley or spectral methods. | RSA is measured as the HRV synchronized with the breathing cycle (inhalation increases heart rate, exhalation decreases it). |
| Root Mean Square of Successive Differences (RMSSD) | A time-domain metric calculated as the square root of the mean of squared differences between successive RR intervals. | Often used to assess parasympathetic activity; sensitive to high-frequency changes (e.g., breathing). |
| Standard Deviation of Successive Differences (SDSD) | The standard deviation of differences between successive RR intervals. | Similar to RMSSD, reflecting parasympathetic modulation. |
| Percentage of NN50 intervals (pNN50) | The percentage of adjacent RR intervals that differ by more than 50 ms. | Indicates parasympathetic activity; sensitive to short-term fluctuations in heart rate. |

***Substance Use.*** Surveys asked participants about substance use occurring the previous day, and participants had 4 hours to complete the surveys. Otherwise, substance use data were collected retrospectively. Compensation was provided based on the number of surveys completed on-time.

***Power Analysis.*** In a cross-over study of patients with schizophrenia, significant increases in glutamate in the hippocampus were found when CBD was administered relative to placebo (d= 0.48; *p*=0.035) [2]. For the fMRI outcomes, to our knowledge, the effects of CBD on cue reactivity have not been assessed using fMRI. However, several studies have demonstrated that CBD can modulate resting-state [4, 5] or task-based [3, 6, 8-12] BOLD signal under similar study design (acute dosing, 600 mg CBD) with sample sizes smaller than our proposed sample.

**
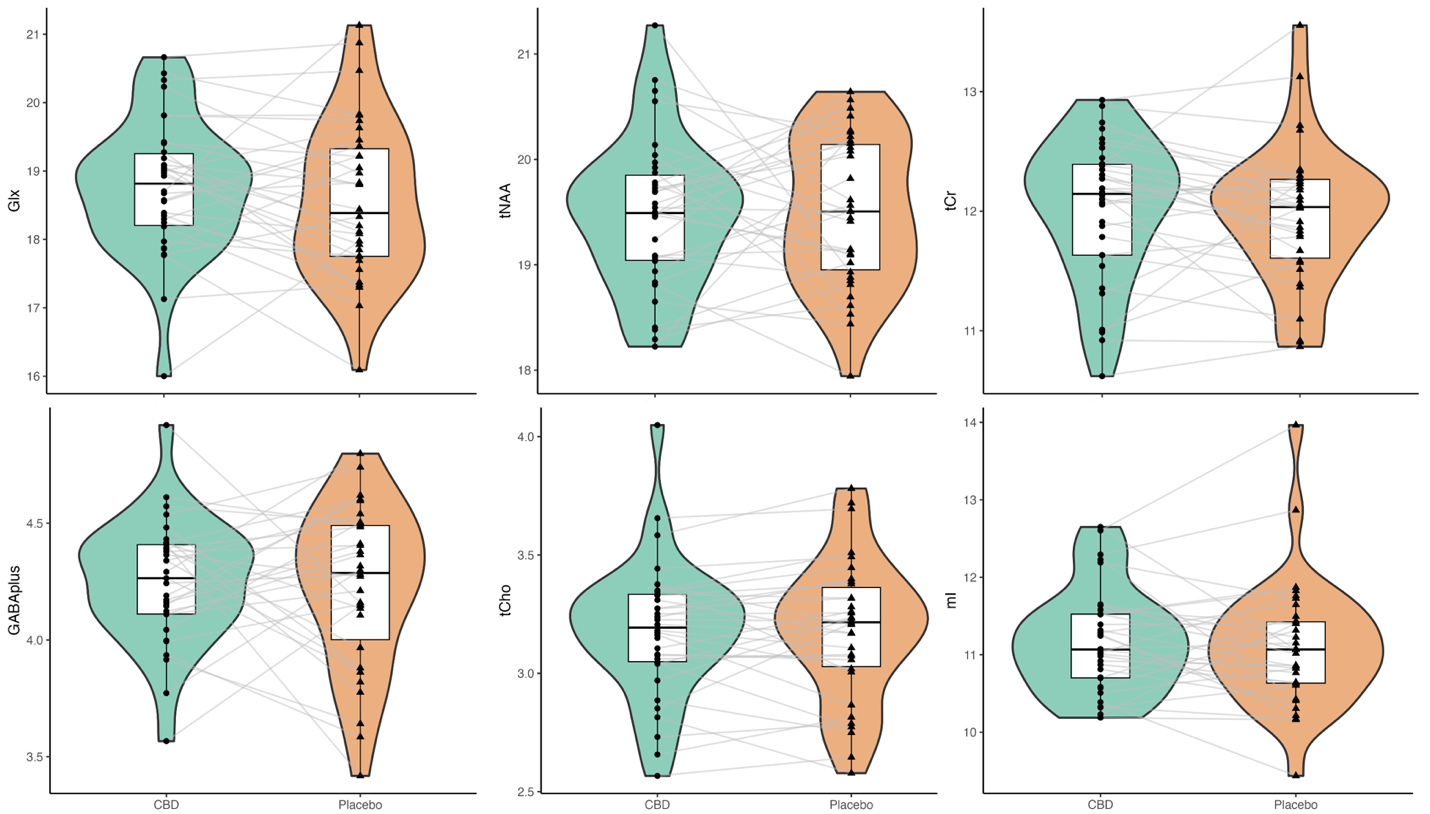
**

**Figure S4.** Neurometabolite Levels (mol/kg) CBD and placebo conditions in the dACC. There were no significant differences between CBD and placebo for any metabolite level. Glx = glutamate + glutamine, GABA+ = GABA plus macromolecules, tNAA = total N-acetylaspartate, tCho = total choline-containing metabolites, tCr = total creatine-containing metabolites, mI = myo-Inositol

**Table S3.** Descriptive Statistics for 1H-MRS Metabolites (n=36).

| **Metabolite** | **Medication** | **Mean** | **St.Dev** | **CoV** |
| --- | --- | --- | --- | --- |
| Glx | CBD | 18.76 | 0.98 | 0.05 |
|  | Placebo | 18.57 | 1.15 | 0.06 |
| tNAA | CBD | 19.48 | 0.72 | 0.04 |
|  | Placebo | 19.50 | 0.70 | 0.04 |
| tCho | CBD | 3.18 | 0.29 | 0.09 |
|  | Placebo | 3.18 | 0.30 | 0.09 |
| tCr | CBD | 12.01 | 0.58 | 0.05 |
|  | Placebo | 11.97 | 0.59 | 0.05 |
| mI | CBD | 11.18 | 0.67 | 0.06 |
|  | Placebo | 11.11 | 0.82 | 0.07 |
| GABAplus | CBD | 4.25 | 0.26 | 0.06 |
|  | Placebo | 4.22 | 0.34 | 0.08 |

Glx = glutamate + glutamine, tNAA = total N-acetylaspartate, tCho = total choline-containing metabolites, tCr = total creatine-containin metabolites, mI = *myo-*Inositol, GABA+ = GABA plus macromolecules; CoV = Coefficient of Variation.

**Table S4.** Tissue Composition and Data Quality Metrics for 1H-MRS Metabolites (n=36).

| **Tissue Type** | **Med** | **Mean** | **St. Dev** | **P-Value** |
| --- | --- | --- | --- | --- |
| fGM* | CBD | 0.59 | 0.03 | 0.63 |
|  | Placebo | 0.59 | 0.03 |  |
| fWM* | CBD | 0.26 | 0.03 | 0.68 |
|  | Placebo | 0.26 | 0.03 |  |
| fCSF* | CBD | 0.15 | 0.02 | 0.99 |
|  | Placebo | 0.15 | 0.03 |  |
| GM:BM* | CBD | 0.69 | 0.03 | 0.62 |
|  | Placebo | 0.70 | 0.04 |  |
| **Data Quality Metric** |  |  |  |  |
| Cr SNR (PRESS)* | CBD | 258.79 | 39.03 | 0.69 |
|  | Placebo | 254.86 | 38.90 |  |
| Cr FWHM (PRESS)** | CBD | 4.67 | 0.57 | 0.05 |
|  | Placebo | 4.72 | 0.37 |  |
| Water FWHM (PRESS)** | CBD | 5.72 | 0.53 | 0.55 |
|  | Placebo | 5.80 | 0.39 |  |
| Cr SNR (MEGA-PRESS) * | CBD | 196.64 | 40.86 | 0.53 |
|  | Placebo | 188.61 | 40.29 |  |
| Cr FWHM (MEGA-PRESS) * | CBD | 4.28 | 0.35 | 0.16 |
|  | Placebo | 4.39 | 0.33 |  |
| Water FWHM (MEGA-PRESS)** | CBD | 5.47 | 0.32 | 0.40 |
|  | Placebo | 5.61 | 0.40 |  |

fGM = fraction of gray matter; fWM = fraction of white matter; fCSF = fraction of cerebral spinal fluid; GM:BM = GM/(GM+WM)]; Cr SNR = Creatine (Cr) signal-to-noise ratio; FWHM = full-width half-maximum

*Student's t-test, **Wilcoxon

**Table S5.** Alcohol Urge Questionnaire (AUQ) Descriptives by Medication and Cue Type (n=36).

| **Medication** | **Cue** | **Mean** | **St.Dev** |
| --- | --- | --- | --- |
| CBD | Alcohol | 23.18 | 11.73 |
|  | Apple Juice | 18.56 | 9.65 |
|  | Baseline | 16.18 | 7.30 |
|  | Water | 16.47 | 7.64 |
| Placebo | Alcohol | 21.40 | 11.22 |
|  | Apple Juice | 17.63 | 10.26 |
|  | Baseline | 14.94 | 6.80 |
|  | Water | 15.50 | 7.14 |

**Table S6.** AUQ Olfactory Cue-Reactivity, Linear Mixed Effects Model (*n*=36)

| Term | Beta | Std. Error | T-Stat | df | P-value |
| --- | --- | --- | --- | --- | --- |
| (Intercept) | 29.60 | 4.49 | 6.59 | 27.61 | 0.00 |
| Medication | -1.53 | 1.20 | -1.27 | 33.68 | 0.21 |
| Sequence | -4.37 | 2.55 | -1.72 | 33.53 | 0.09 |
| Visit | -0.21 | 0.96 | -0.22 | 30.16 | 0.83 |
| Cue (Apple Juice) | -4.62 | 1.12 | -4.11 | 33.00 | **<0.001** |
| Cue (Baseline) | -7.00 | 1.42 | -4.92 | 33.00 | **<0.001** |
| Cue (Water) | -6.71 | 1.32 | -5.06 | 33.00 | **<0.001** |
| Medication*Cue (Apple Juice) | 0.85 | 1.40 | 0.60 | 34.25 | 0.55 |
| Medication*Cue (Baseline) | 0.54 | 1.09 | 0.50 | 34.25 | 0.62 |
| Medication*Cue (Water) | 0.53 | 1.00 | 0.53 | 34.19 | 0.60 |

Note: AUQ model used robust errors; Medication defined as CBD (reference) or placebo; Sequence defined as CBD/Placebo (reference) or Placebo/CBD; Visit defined as neuroimaging Visit 1 (reference) or Visit 2

**Table S7.** Heart Rate Variability (HRV) Descriptives by Medication (n= 36)

| **HRV Outcome** | **Medication** | **Mean** | **St.Dev** |
| --- | --- | --- | --- |
| Sympathetic | CBD | 0.38 | 0.10 |
|  | Placebo | 0.37 | 0.11 |
| Vagal | CBD | 0.62 | 0.10 |
|  | Placebo | 0.63 | 0.11 |
| Sympathetic: Vagal | CBD | 0.84 | 0.37 |
|  | Placebo | 0.81 | 0.41 |
| RSA | CBD | 8.56 | 1.65 |
|  | Placebo | 8.46 | 1.52 |
| RMSSD | CBD | 433.08 | 970.86 |
|  | Placebo | 109.62 | 120.05 |
| SDSD | CBD | 380.17 | 868.16 |
|  | Placebo | 109.60 | 120.05 |
| pNN50 | CBD | 33.53 | 22.19 |
|  | Placebo | 32.50 | 22.48 |

Note: Data over the full olfactory cue-reactivity task, not related to cues

RSA = Respiratory Sinus Arrhythmia; RMSSD = Root Mean Square of Successive Differences; SDSD = Standard Deviation of Successive Differences; pNN50 = Percentage of NN50 intervals

**Table S8.** HRV Outcomes for Full Olfactory Cue-Reactivity Task, Linear Mixed Effects Model (n=36)

| HRV Outcome | Term | Beta Estimate | Std. Error | T-Stat | df | P-value |
| --- | --- | --- | --- | --- | --- | --- |
| Sympathetic | (Intercept) | 0.42 | 0.06 | 6.67 | 18.15 | 0.00 |
|  | Medication | -0.01 | 0.02 | -0.51 | 30.17 | 0.61 |
|  | Sequence | 0.00 | 0.03 | -0.12 | 22.73 | 0.90 |
|  | Visit | -0.02 | 0.02 | -1.22 | 29.92 | 0.23 |
|  | 1-Year Moderate AUD | -0.06 | 0.04 | -1.75 | 16.34 | 0.10 |
|  | 1-Year Severe AUD | 0.06 | 0.04 | 1.62 | 8.91 | 0.14 |
| Vagal | (Intercept) | 0.58 | 0.06 | 9.05 | 18.15 | 0.00 |
|  | Medication | 0.01 | 0.02 | 0.51 | 30.17 | 0.61 |
|  | Sequence | 0.00 | 0.03 | 0.12 | 22.73 | 0.90 |
|  | Visit | 0.02 | 0.02 | 1.22 | 29.92 | 0.23 |
|  | 1-Year Moderate AUD | 0.06 | 0.04 | 1.75 | 16.34 | 0.10 |
|  | 1-Year Severe AUD | -0.06 | 0.04 | -1.62 | 8.91 | 0.14 |
| Sympathetic: Vagal | (Intercept) | 1.00 | 0.23 | 4.32 | 19.27 | 0.00 |
|  | Medication | -0.02 | 0.08 | -0.23 | 30.80 | 0.82 |
|  | Sequence | -0.04 | 0.11 | -0.41 | 22.40 | 0.69 |
|  | Visit | -0.07 | 0.08 | -0.92 | 30.51 | 0.37 |
|  | 1-Year Moderate AUD | -0.17 | 0.13 | -1.31 | 15.95 | 0.21 |
|  | 1-Year Severe AUD | 0.28 | 0.13 | 2.15 | 8.91 | 0.06 |
| RSA | (Intercept) | 7.79 | 0.98 | 7.93 | 16.66 | 0.00 |
|  | Medication | -0.25 | 0.24 | -1.05 | 29.29 | 0.30 |
|  | Sequence | 0.37 | 0.54 | 0.68 | 23.06 | 0.50 |
|  | Visit | 0.36 | 0.24 | 1.53 | 29.13 | 0.14 |
|  | 1-Year Moderate AUD | 0.01 | 0.51 | 0.02 | 16.69 | 0.98 |
|  | 1-Year Severe AUD | -1.01 | 0.63 | -1.59 | 8.91 | 0.15 |
| RMSSD* | (Intercept) | 43.55 | 30.18 | 1.44 | 25.87 | 0.16 |
|  | Medication | -3.37 | 12.27 | -0.27 | 24.37 | 0.79 |
|  | Sequence | 24.72 | 18.10 | 1.37 | 28.82 | 0.18 |
|  | Visit | 4.72 | 12.27 | 0.38 | 24.37 | 0.70 |
| SDSD* | (Intercept) | 43.54 | 30.18 | 1.44 | 25.87 | 0.16 |
|  | Medication | -3.37 | 12.26 | -0.27 | 24.37 | 0.79 |
|  | Sequence | 24.72 | 18.10 | 1.37 | 28.82 | 0.18 |
|  | Visit | 4.71 | 12.26 | 0.38 | 24.37 | 0.70 |
| PNN50 | (Intercept) | 15.96 | 11.31 | 1.41 | 27.72 | 0.17 |
|  | Medication | -2.12 | 3.30 | -0.64 | 29.39 | 0.53 |
|  | Sequence | 11.49 | 7.02 | 1.64 | 33.25 | 0.11 |
|  | Visit | 0.97 | 3.30 | 0.29 | 29.39 | 0.77 |

Note: all models used robust errors; * 7 outliers removed (n=5 for CBD, n=2 for placebo)

RSA = respiratory sinus arrhythmia; RMSSD = root mean square of successive differences; SDSD= standard deviation of successive differences; pNN50 = percentage of NN50 intervals; Medication defined as CBD (reference) or placebo; Sequence defined as CBD/Placebo (reference) or Placebo/CBD; Visit defined as neuroimaging Visit 1 (reference) or Visit 2; 1-Year; 1-Year AUD Severity defined by number of AUD symptoms in the past year on the MINI at screening (2-3 = mild [reference], 4-5 = moderate, 6 or more = severe).


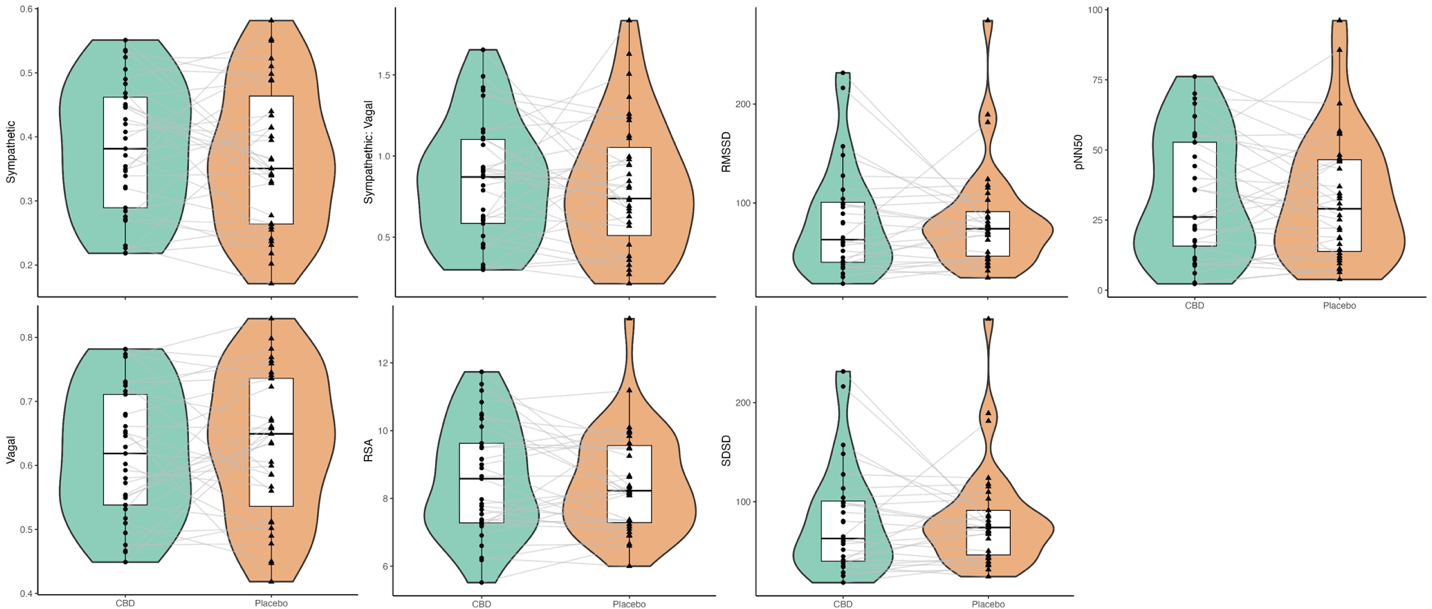


**Figure S5.** Heart Rate Variability (HRV) Outcomes for CBD and placebo conditions. There were no significant differences between CBD and placebo for any HRV outcome. RSA = respiratory sinus arrhythmia; RMSSD = root mean square of successive differences; SDSD= standard deviation of successive differences; pNN50 = percentage of NN50 intervals

**Table S9.** Skin Conductance Response (SCR) Descriptives by Medication (n=28)

| **SCR Outcome** | **Medication** | **Mean** | **St. Dev** | **Min** | **Max** |
| --- | --- | --- | --- | --- | --- |
| Number of SCRs | CBD | 52.06 | 72.52 | 0.00 | 312.00 |
|  | Placebo | 39.57 | 55.24 | 0.00 | 252.00 |
| Mean Amplitude | CBD | 4.65 | 4.81 | 0.00 | 19.63 |
|  | Placebo | 3.73 | 3.87 | 0.00 | 14.91 |
| Median Amplitude | CBD | 4.49 | 4.40 | 0.00 | 17.71 |
|  | Placebo | 3.77 | 3.99 | 0.00 | 15.62 |
| St. Dev Amplitude | CBD | 1.19 | 2.10 | 0.00 | 9.04 |
|  | Placebo | 0.60 | 0.67 | 0.00 | 2.32 |
| Peak Intervals | CBD | 6.42 | 7.06 | 0.00 | 30.22 |
|  | Placebo | 5.01 | 5.23 | 0.00 | 19.15 |

Note: Data over the full olfactory cue-reactivity task, not related to cues

**Table S10.** SCR Outcomes for Full Olfactory Cue-Reactivity Task, Linear Mixed Effects Model (n=28)

| SCR Outcome | Term | Beta Estimate | Std. Error | T-Stat | df | P-value |
| --- | --- | --- | --- | --- | --- | --- |
| Number of SCRs | (Intercept) | 29.93 | 11.65 | 2.57 | 23.44 | 0.02 |
|  | Medication | 8.09 | 5.49 | 1.47 | 24.53 | 0.15 |
|  | Sequence | 1.81 | 6.38 | 0.28 | 27.64 | 0.78 |
|  | Visit | -8.39 | 5.49 | -1.53 | 24.53 | 0.14 |
| Mean Amplitude | (Intercept) | 7.13 | 3.05 | 2.34 | 25.35 | 0.03 |
|  | Medication | -0.86 | 0.74 | -1.16 | 21.11 | 0.26 |
|  | Sequence | -0.61 | 1.30 | -0.47 | 30.32 | 0.64 |
|  | Visit | -1.29 | 0.74 | -1.73 | 21.11 | 0.10 |
| Median Amplitude | (Intercept) | 6.43 | 2.69 | 2.39 | 25.45 | 0.02 |
|  | Medication | -0.63 | 0.71 | -0.89 | 20.98 | 0.38 |
|  | Sequence | -0.45 | 1.24 | -0.36 | 30.41 | 0.72 |
|  | Visit | -1.12 | 0.71 | -1.57 | 20.98 | 0.13 |
| St. Dev Amplitude | (Intercept) | 3.15 | 1.59 | 1.97 | 20.41 | 0.06 |
|  | Medication | -0.63 | 0.39 | -1.64 | 24.67 | 0.11 |
|  | Sequence | -0.69 | 0.45 | -1.54 | 27.76 | 0.13 |
|  | Visit | -0.63 | 0.39 | -1.64 | 24.67 | 0.11 |
| Peak Interval | (Intercept) | 10.52 | 4.62 | 2.28 | 25.09 | 0.03 |
|  | Medication | -1.52 | 1.12 | -1.36 | 21.45 | 0.19 |
|  | Sequence | -1.25 | 1.83 | -0.68 | 30.10 | 0.50 |
|  | Visit | -1.79 | 1.12 | -1.59 | 21.45 | 0.13 |

Note: all models used robust errors; Medication defined as CBD (reference) or placebo; Sequence defined as CBD/Placebo (reference) or Placebo/CBD; Visit defined as neuroimaging Visit 1 (reference) or Visit 2


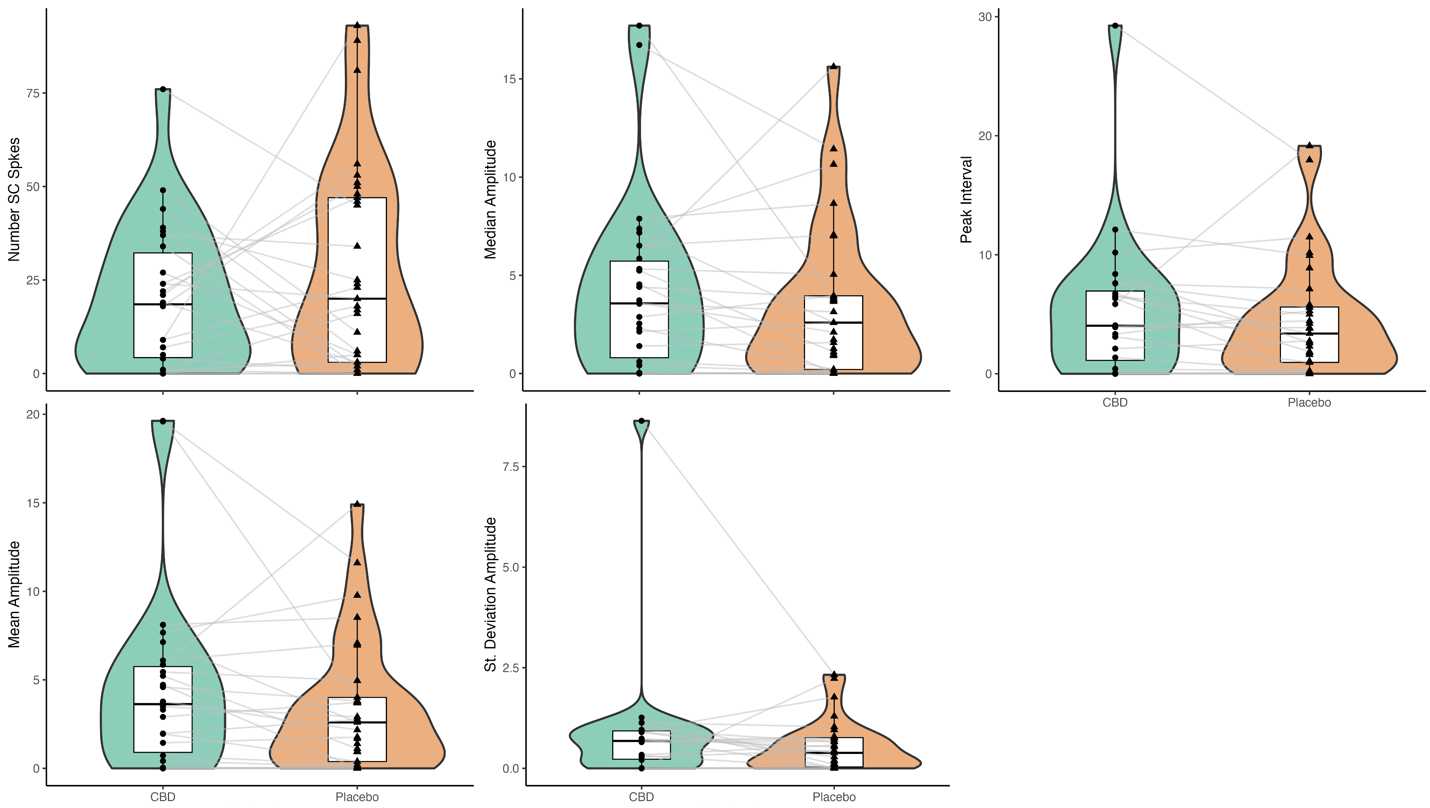


**Figure S6.** Skin Conductance Outcomes for CBD and placebo conditions. There were no significant differences between CBD and placebo for any HRV outcome.

**Table S11.** Daily Diary Descriptives by Medication (n=35 participants, 466 diaries)

| **Alcohol Outcome** | **Medication** | **Percent** | **Median** | **Min** | **Max** |
| --- | --- | --- | --- | --- | --- |
| Drinking days | CBD | 30.13% | 2 | 0 | 6 |
|  | Placebo | 30.80% | 2 | 0 | 7 |
| Binge drinking days | CBD | 15.28% | 1 | 0 | 3 |
|  | Placebo | 12.24% | 1 | 0 | 3 |
|  | **Medication** | **Mean** | **St. Dev** | **Min** | **Max** |
| Drinks per day | CBD | 1.43 | 3.00 | 0.00 | 19.00 |
|  | Placebo | 1.28 | 2.58 | 0.00 | 16.00 |
| Drinks per drinking day | CBD | 4.76 | 3.76 | 1.00 | 19.00 |
|  | Placebo | 4.16 | 3.10 | 1.00 | 16.00 |

Note. Descriptives include the 7 days following each acute medication administration,

excluding missing data. Median, min, and max for binge drinking days and drinking days represent participant-level counts

**Table S12.** Daily Drinks, Zero-Inflated Negative Binomial Mixed Effects Model (*n*=35, obs=466)

| **Conditional Model Terms:** | **Beta** | **Std. Error** | **Z-value** | **P-value** |
| --- | --- | --- | --- | --- |
| (Intercept) | 0.81 | 0.21 | 3.79 | **<0.001** |
| Medication | -0.15 | 0.14 | -1.08 | 0.28 |
| Sequence | 0.18 | 0.16 | 1.15 | 0.25 |
| Visit | 0.18 | 0.14 | 1.29 | 0.20 |
| Baseline drinks per drinking day | 0.10 | 0.03 | 3.81 | **<0.001** |
| **Zero-Inflation Model:** | **Beta** | **Std. Error** | **Z-value** | **P-value** |
| (Intercept) | 0.68 | 0.11 | 5.94 | **<0.0001** |

Note: Outcome, daily drinks, covers the 7 days following each acute medication administration; Medication defined as CBD (reference) or placebo; Sequence defined as CBD/Placebo (reference) or Placebo/CBD; Visit defined as Visit 1 (reference) or Visit 2; Baseline drinks per drinking day defined as the average drinks per drinking day from 60-days TLFB at screening.

1. Pretzsch, C.M., et al., *Effects of cannabidiol on brain excitation and inhibition systems; a randomised placebo-controlled single dose trial during magnetic resonance spectroscopy in adults with and without autism spectrum disorder.* Neuropsychopharmacology, 2019. **44**(8): p. 1398-1405.

2. O’Neill, A., et al., *Cannabidiol modulation of hippocampal glutamate in early psychosis.* Journal of Psychopharmacology, 2021: p. 02698811211001107.

3. O'Neill, A., et al., *Normalization of mediotemporal and prefrontal activity, and mediotemporal-striatal connectivity, may underlie antipsychotic effects of cannabidiol in psychosis.* Psychological Medicine, 2020: p. 1-11.

4. Grimm, O., et al., *Probing the endocannabinoid system in healthy volunteers: Cannabidiol alters fronto-striatal resting-state connectivity.* European Neuropsychopharmacology, 2018. **28**(7): p. 841-849.

5. Pretzsch, C.M., et al., *The effect of cannabidiol (CBD) on low-frequency activity and functional connectivity in the brain of adults with and without autism spectrum disorder (ASD).* Journal of Psychopharmacology, 2019. **33**(9): p. 1141-1148.

6. Davies, C., et al., *A single dose of cannabidiol modulates medial temporal and striatal function during fear processing in people at clinical high risk for psychosis.* Translational psychiatry, 2020. **10**(1): p. 1-12.

7. Taylor, L., et al., *A phase I, randomized, double-blind, placebo-controlled, single ascending dose, multiple dose, and food effect trial of the safety, tolerability and pharmacokinetics of highly purified cannabidiol in healthy subjects.* CNS drugs, 2018. **32**(11): p. 1053-1067.

8. Bhattacharyya, S., et al., *Opposite effects of Δ-9-tetrahydrocannabinol and cannabidiol on human brain function and psychopathology.* Neuropsychopharmacology, 2010. **35**(3): p. 764-774.

9. Winton-Brown, T.T., et al., *Modulation of auditory and visual processing by delta-9-tetrahydrocannabinol and cannabidiol: an FMRI study.* Neuropsychopharmacology, 2011. **36**(7): p. 1340-1348.

10. Borgwardt, S.J., et al., *Neural basis of Δ-9-tetrahydrocannabinol and cannabidiol: effects during response inhibition.* Biological psychiatry, 2008. **64**(11): p. 966-973.

11. Fusar-Poli, P., et al., *Distinct effects of Δ9-tetrahydrocannabinol and cannabidiol on neural activation during emotional processing.* Archives of general psychiatry, 2009. **66**(1): p. 95-105.

12. Bhattacharyya, S., et al., *Induction of psychosis byδ9-tetrahydrocannabinol reflects modulation of prefrontal and striatal function during attentional salience processing.* Archives of general psychiatry, 2012. **69**(1): p. 27-36.
